# Understanding the transgression of global and regional freshwater planetary boundaries

**DOI:** 10.1098/rsta.2021.0294

**Published:** 2022-12-12

**Authors:** A. V. Pastor, H. Biemans, W. Franssen, D. Gerten, H. Hoff, F. Ludwig, P. Kabat

**Affiliations:** ^1^ ITAP, Univ Montpellier, INRAE, Institut Agro, Montpellier, France; ^2^ Elsa, Research Group for Environmental Lifecycle and Sustainability Assessment, Montpellier, France; ^3^ Water Systems and Global Change Group, Wageningen University, Wageningen UR, PO Box 47, Wageningen 6700 AA, The Netherlands; ^4^ Water and Food research group, Wageningen University and Research, Wageningen, The Netherlands; ^5^ Research Domain of Earth System Analysis, Potsdam Institute for Climate Impact Research (PIK), Telegraphenberg A62, Potsdam 14473, Germany; ^6^ Geography Department, Humboldt-Universität zu Berlin, Unter den Linden 6, Berlin 10099, Germany; ^7^ Stockholm Environment Institute, Kräftriket 2b, Stockholm SE-106 91, Sweden; ^8^ World Meteorological Organization (WMO), 7bis Avenue de la Paix Case postale 2300 Nations, Genève 1211, Suisse

**Keywords:** freshwater planetary boundaries, environmental flow, water deficit, water abstraction, water stress index

## Abstract

Freshwater ecosystems have been degraded due to intensive freshwater abstraction. Therefore, environmental flow requirements (EFRs) methods have been proposed to maintain healthy rivers and/or restore river flows. In this study, we used the Variable Monthly Flow (VMF) method to calculate the transgression of freshwater planetary boundaries: (1) natural deficits in which flow does not meet EFRs due to climate variability, and (2) anthropogenic deficits caused by water abstractions. The novelty is that we calculated spatially and cumulative monthly water deficits by river types including the frequency, magnitude and causes of environmental flow (EF) deficits (climatic and/or anthropogenic). Water deficit was found to be a regional rather than a global concern (less than 5% of total discharge). The results show that, from 1960 to 2000, perennial rivers with low flow alteration, such as the Amazon, had an EF deficit of 2–12% of the total discharge, and that the climate deficit was responsible for up to 75% of the total deficit. In rivers with high seasonality and high water abstractions such as the Indus, the total deficit represents up to 130% of its total discharge, 85% of which is due to withdrawals. We highlight the need to allocate water to humans and ecosystems sustainably.

This article is part of the Royal Society Science+ meeting issue ‘Drought risk in the Anthropocene’.

## 1. Introduction

In the past decades, increased human water use has resulted in altered flows in major river basins such as the Indus and the Colorado Rivers, with 30% overuse of non-renewable water resources [1,2]. Flow alteration and the induced loss of river connectivity are the primary causes of freshwater ecosystem damage [3,–5]. There is a growing concern about the decline of natural resources, especially the loss of freshwater species [6,7]. Maintaining rivers close to their natural flow regime is necessary to fulfil ecosystem functions [8]. Recent studies show the impact of multiple stressors on the ecological status of global and European rivers [9,–12]. To limit and reduce the degradation of freshwater ecosystems, river restoration projects have emerged to restore freshwater ecosystems to acceptable ecological conditions.

To maintain rivers in ‘acceptable’ ecological conditions, environmental flow requirements (EFRs) have been defined as ‘the quantity, timing and quality of freshwater flows and levels necessary to sustain aquatic ecosystems which, in turn, support human cultures, economies, sustainable livelihoods and well-being’ [13,14]. Recent studies have assessed the transgressions of freshwater planetary boundaries [15,–18], but still calculating the deficit over an averaged period without thorough evaluation. Indeed and to our knowledge, there is no study that looked at EF deficit (also defined here as water planetary boundary (PB) transgression) at such spatial (country, river basin and global) and time (monthly including the frequency (number of months and duration)) resolutions. Also, there is no study that differentiated whether environmental flow (EF) deficit occurred due to climate (natural deficit) and/or water abstraction (anthropogenic deficit). By differentiating whether water deficit is due to water abstract versus climatic conditions we can allow water managers to anticipate whether water restrictions should be more focused on building water reservoirs, and/or restricting temporarily water access.

In this study, we adapted the concept of ‘eco-deficit’ defined by Vogel *et al*. [19] to the concept of ‘environmental flow deficit’ to show in time and space the water flow deficit in a river on an intra-annual basis (e.g. seasonal water deficit) and on an inter-annual basis (e.g. supra-seasonal water deficit). We have adapted this concept to differentiate the cumulative water deficit due to climate variability and/or water abstractions. Extreme events such as meteorological droughts can cause hydrological droughts in which ecosystems have a margin of manoeuvre to adapt and survive [20]. However, if the deficit is redundant, it can be detrimental to freshwater ecosystems and lead to their collapse. Therefore, it is important to differentiate the frequency, magnitude and causes of EF deficits for different flow regimes (perennial, intermittent). While perennial rivers are known to have a stable flow throughout the year, intermittent rivers are characterized by a period of flow cessation [21].

On one side, the water deficit (caused by climate variability such as prolonged drought) is not always detrimental to freshwater ecosystems. For example, some species have become resilient to periodic droughts [22]. In some cases, extreme events may even be required to remove invasive species [23,24]. Natural disturbances such as floods and drought events can also help regulate population size and species diversity [25]. Recovery of freshwater species is linked to three types of adaptive mechanisms: life history, behaviour and morphology, which are used by species to cope with natural disturbances. However, a long-term excessive deficit could lead to species extinction due to poor provision of refugia for species recovery, and subsequent abrupt changes in biological community structure and ecosystem processes [26]. Therefore, natural deficit may have a greater impact on ecosystems with climate change. On the other side, anthropogenic deficit (caused by water abstraction for irrigation, industry and households) may lead to a shift in flow regime jeopardizing the natural integrity of freshwater ecosystems [27]. Anthropogenic deficit can also exacerbate the effect of drought and water deficit on freshwater ecosystems by ‘reducing the resistance and the resilience of species' [23]. For example, important components of the flow in the Murray-Darling river, such as low flow duration, were shown to be heavily modified after water abstraction [22,28]. Besides, increasing the duration of low flows can reduce riparian vegetation and lead to physiological stress [29]. Furthermore, dams and reservoirs can harm freshwater ecosystems by disrupting the existing movement of water and sediments that provide food and refugia for most river taxa [27]. In this study, we calculated the water deficit at the global level and for 23 major river basins.

Our objectives were to (i) differentiate between natural and anthropogenic EF deficits, (ii) define EF deficits on a global scale in terms of intra-annual and inter-annual variability, (iii) deepen the understanding of EF deficits in terms of timing, frequency and duration, (iv) categorize river basins in terms of flow regime, flow alteration and sensitivity to EF deficits and lastly (v) compute global water stress taking into EFRs.

## 2 Material and methods

### (a) Model

We used the global dynamic vegetation and hydrological model LPJmL [30,–33] to simulate natural discharge (without anthropogenic water use) and actual discharge (including anthropogenic water use for irrigation, industries and households). LPJmL was developed to consistently simulate global water and carbon cycles for natural and agricultural vegetation. LPJmL calculates daily water budgets at 0.5-degree spatial resolution with flow components routed laterally along with the Simulated Topological Network (STN-30) with a constant flow velocity of 1 ms^−1^ [34]. Runoff occurs when water input exceeds soil water capacity in a multi-layer soil column, which is routed downstream according to the topology. For details on the simulation of crop growth and irrigation requirements, please refer to Rost *et al*. [32]. Each cell containing crops and pasture can be irrigated and/or rain-fed, based on a map of irrigated areas [35]. Water is extracted from surface water including rivers, dams and reservoirs for irrigation and other uses. The estimated volume of water withdrawn for irrigation is calculated based on the specific daily demand of each crop [34]. Industrial and household water use is also included in the simulation and estimated between 750 and 1000 km^3^ yr^−1^ from 1960 to 2000 [36]. Conveyance losses, which represent the volume of water lost during transport, and irrigation use efficiency are included in the irrigation calculations to provide gross irrigation demand [32]. Net irrigation requirements are computed at daily time resolution and 0.5° spatial resolution based on evaporation demand and soil water capacity requirements. Irrigation withdrawals were calculated assuming that any irrigation requirement can be met [32,34]. Within a river basin, a calculation was performed so that runoff and EFRs were re-allocated according to discharge repartition [37].

**Figure 1.**
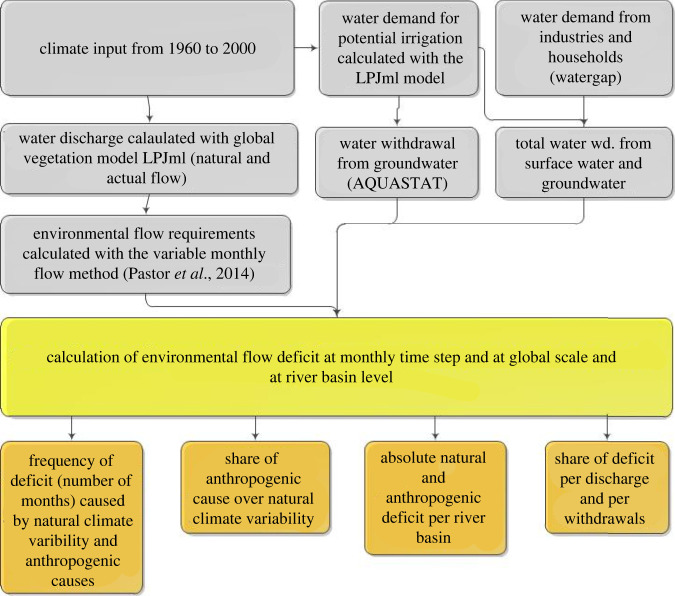
Conceptual framework to calculate EFR deficit on the global scale. (Online version in colour.)

Irrigation withdrawals can be simulated with four conditions: under unlimited water supply (IPOT), here water withdrawals equal water demands for irrigation use and the water that is not available from surface water is supposed to come from non-renewable sources such as groundwater. The second irrigation simulation is based on water availability from rivers, dams and reservoirs (IRES) only, so that if water demand exceeds supply, plant growth will be limited to water supply [32,34]. Third, runoff can also be simulated under natural conditions without irrigation withdrawals (INO) where all crops are rainfed. Fourth, runoff can also be simulated with natural vegetation only (PNV) without irrigation. Potential evapotranspiration (PET) is calculated with the Priestley-Taylor method using soil moisture and rooting depth information, while actual evapotranspiration (ET) is based on PET and potential canopy conductance (calculated with photosynthesis and CO_2_ concentrations) [30,31]. In this study, we used fixed land use in the year 2000 to evaluate the impact of inter-annual climate variability. In this study, we used different irrigation simulations for different purposes. The IPOT simulation was used to calculate total irrigation requirements based on PET. In that simulation, water can come from various sources (surface water and groundwater). The IRES simulation is used to calculate the amount of water used from surface water for irrigation. The difference between IPOT and IRES in terms of irrigation requirements is usually equivalent to water coming from other sources (mostly groundwater). Finally, we used the INO simulation to simulate all crops as rainfed (without any water infrastructures). We forced the model with the input climate dataset CRU T.S.3.10 (available online at http://badc.nerc.ac.uk/data/cru/). The input consists of monthly precipitation values, number of wet days, cloud cover fraction and average temperature for 41 years (1960–2000). The model was first run with a 1000-year spin-up to bring the carbon and water cycles in equilibrium. Then, we ran the model for 41 years (1960–2000) to include inter-annual variability in our analyses. Regarding model performance, validation of major river basins and crop yields was performed [11,17,34] with very satisfactory correlations (*R*^2^ > 0.6) [11,34]. The conceptual framework of the study is presented figure 1.

In this study, we have focused our results on one GHM which is LPJml because this model has proven to have good performances and is the closest to the ensemble mean for runoff in [38]. We have also used one climatic dataset CRU which is based on global observed runoff. This was also the case in a previous study [11].

### (b) Environmental flow method

In this study, we use the variable monthly flow (VMF) method [39] to calculate environmental flow requirements (EFRs) globally with the INO simulation. This method is widely applied in environmental assessments [11,15,40–42]. Each grid cell has a specific seasonal flow regime (hydrograph), which was divided into three periods: low-flow, high-flow and intermediate-flow periods. High flows are assigned when the mean monthly flow (MMF) is above 80% of the mean annual flow (MAF); low flows are assigned when the MMF is below 40% of the MAF; intermediate flows are assigned when the MMF is between 40% and 80% of the MAF. We then allocate 30% of the MMF for high-flow requirements, 45% of the MMF for intermediate-flow requirements and 60% of the MMF for low-flow requirements. In this study, we define net discharge as the total discharge minus EFRs. We used the VMF method because it is a global EF method with high spatial and temporal resolutions that were validated with local study cases [39]. Moreover, it has been widely implemented and accepted in the latest global assessments [12,15,16,40,41,43]. It is also important to give a range of EFRs to water managers to allow some flexibility (this study and in [44]). Other studies providing sensitivity analyses of EF methods show small differences between methods on water resources and food production and a lower impact on the latter than with drivers such as climate change and/or population increase [11,15,45].

### (c) Planetary boundary transgressions and definition of EF deficit

Planetary boundary transgressions occur any time in space and time EF deficit is occurring. We define two types of EF deficits: the deficit coming from climate variability and the deficit coming from water abstraction. By using monthly flow data, we define the intra-annual deficit to identify the timing and duration of the deficit. We also calculate the frequency of natural deficit by using the number of months and years. To calculate the anthropogenic deficit at global scale, we differentiate water withdrawals from surface water and groundwater. Groundwater withdrawals account for 851 km^3^ yr^−1^. and were calculated as a share of total potential withdrawals per country [46]. The EF deficit is calculated on the grid cell and was aggregated at the river basin or country scale depending on the analysis.

EF deficits are defined below:

2.1 EF_def_nat(m,y)=∑i=1j=1y,m(Qnat(m,y)−EFR(m))

2.2 EF_def_ant(m,y)=∑i=1j=1y,m(Qmod(m,y)−EFR(m))

where EF_def_nat represents the deficit coming from climate and EF_def_ant represents the deficit coming from water abstraction. Qnat represents the natural discharge and Qmod represents the current modified discharge including water withdrawals. *y* represents the number of years (*y* = 41) to iterate starting from *i*. *m* represents the number of months (*m* = 12) to iterate starting from *j*. EFR represents monthly environmental flow. As defined in Steffen *et al*. [15], the transgression of environmental flow requirements is used to define the freshwater planetary boundaries.

### (d) Sensitivity analyses and statistics

For sensitivity analyses and uncertainty range, we calculated deficits for two levels of EFRs: normal (VMF method) and high (VMF method + 1 s.d., see [44]). The standard deviation of EFRs was calculated over 41 years. The maximum, minimum, standard deviation of hydrological indexes were calculated to give ranges in table 1 and supplementary table S1.

### (e) Classification of river basins

We use principal component analysis (PCA) to identify river basins categories using six hydrological variables. Explained variance is a statistical measure of how much variation in a dataset can be attributed to each of the principal components (eigenvectors) generated by a PCA. These hydrological variables were chosen to define river flow regimes, the level of flow alteration, differentiation between anthropogenic and natural deficit and finally to define the duration, timing and frequency of deficit. Principal component analysis of a data matrix allows the extraction of the main patterns of the matrix in terms of a corresponding set of score and load plots [47]. We selected hydrological indicators for which the input to the PCA had the highest explained variance. We selected 23 river basins worldwide to represent three different river flow regimes (perennial, intermittent and erratic), two different climates (tropical and temperate) and two levels of flow alteration (below 50% alteration and above 50% of flow alteration).

To characterize the flow regime, we used two variables: the base flow index (BFI) [48] and the hydrological variability index (HVI) variables were defined as below:

2.3 $$\text{BFI}=\;\frac{\text{Q}90}{\text{MAF}}$$

2.4 $$\text{HVI}=\frac{\text{Q}25-\text{Q}75}{\text{Q}50}$$

where Q90 represents the flow which is exceeded for 90% of the period of record; MAF represents the mean annual flow; Q25 represents the flow which is exceeded for 25% of the period of record; Q75 represents the flow which is exceeded for 75% of the period of record, and Q50 represents the flow which is exceeded for 50% of the period of record. All our calculations are based on monthly output where volumes of water are in km^3^. MOD represents the level of flow modification and is defined as the mean annual actual flow (including anthropogenic water abstractions) over the mean annual natural flow. Therefore, if MOD is close to 1, it means it is almost natural and barely modified.

To perform the PCA, 11 hydrological indicators were used to define the range of origin, magnitude, timing and frequency of the EF deficit:

— Total deficit to discharge
  2.5 DTD(m,y)=∑i=1j=1y,mNat. Deficit (m,y)+Ant. Deficit (y,m)Qnat (m,y)
— Natural deficit to discharge
  2.6 DTN(m,y)=∑i=1j=1y,mNat. Deficit (m,y)Qnat (m,y)
— Anthropogenic deficit to discharge
  2.7 DTA(m,y)=∑i=1j=1y,mAnt. Deficit (m,y)Qnat (m,y)
— Frequency of total deficit – sum of the number of months deficit
  2.8 $$\text{Freq}\_\text{tot}(y)=\sum_{j=1}^{y}\text{ME}{\text{F}}_{\text{def}1(y)}+\text{ME}{\text{F}}_{\text{def}2(y)}$$
— Frequency of natural deficit – sum of the number of months deficit
  2.9 $$\text{Freq}\_\text{nat}(y)=\sum_{j=1}^{y}\text{ME}{\text{F}}_{\text{def}1(y)}$$
— Frequency of anthropogenic deficit – sum of the number of months deficit
  2.10 Freq_ant(m,y)=∑i=1j=1y,mMEFdef2(m,y)
— Ratio of the anthropogenic deficit over natural deficit
  2.11 ATN(m,y)=∑i=1j=1y,mAnt. Deficit (m,y)Nat. Deficit (m,y)
— Ratio of the frequency anthropogenic deficit over natural deficit
  2.12 FATN(m,y)=∑i=1j=1y,mFreq_ant. (m,y)Freq_nat. (m,y)
— Ratio of water withdrawals over discharge
  2.13 WTD(m,y)=∑i=1j=1y,mWd (m,y)Qnat (m,y)
— Ratio of the deficit over water withdrawals
  2.14 DTW(m,y)=∑i=1j=1y,mEFdef1,2 (m,y)Wd (m,y)
— Ratio of natural deficit over water withdrawals
  2.15 NTW(m,y)=∑i=1j=1y,mEFdef1 (m,y)Wd (m,y)
— Ratio of the anthropogenic deficit over water withdrawals coming from surface water
  2.16 ATWs(m,y)=∑i=1j=1y,mEFdef1 (m,y)Wd_surf (m,y)

where MEF represents months with the environmental flow deficit with def1 representing the natural deficit and def2 the anthropogenic deficit, Wd and Wd_surf represent respectively the total water withdrawals coming from surface water only and where m and y represent months and years.

Finally, the water stress indicator (WSI) was defined such as in [49]. WSI is calculated with and without EFR.

2.17 WSI(m,y)=∑i=1j=1y,mWd(m,y)(+EFRs)Water available (m,y)

If WSI exceeds 100% of discharge, the basin is classified as ‘environmentally water scarce’. In this case, total withdrawals are outpacing total surface water availability so that there is no water left for EFRs. Basins where 60% < WSI < 100% are defined as ‘environmentally water-stressed’ and basins where 30% < WSI < 60% as moderately exploited. Finally, when WSI < 30%, the basins are defined as ‘environmentally safe’. All the abbreviations are found in the electronic supplementary material.

## 3. Results

### (a) Global water deficit

**Figure 2.**
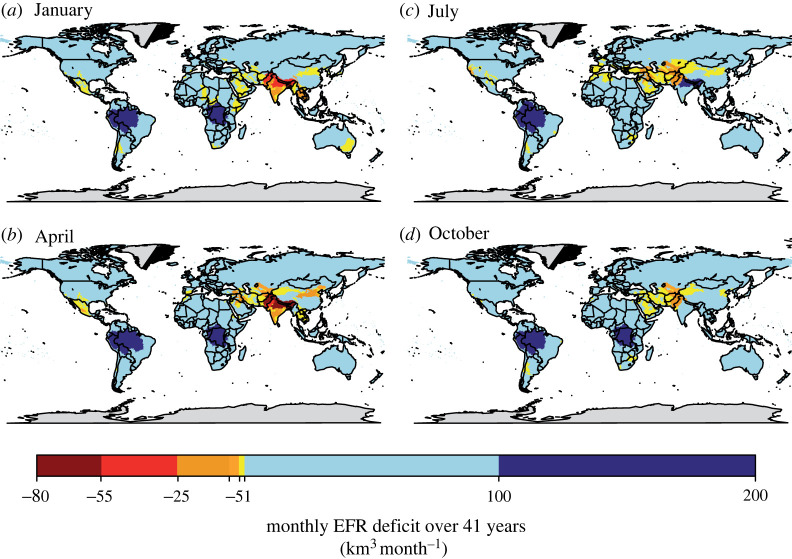
Spatial representation of monthly deficit on the global scale for four months: Jan, Apr, Jul, Oct averaged over 41 years. Units are in km^3^ month^−1^. Negative values represent deficit of flow (in yellow/orange/red) whereas positive values (in blue) represent surpluses of flow. Light blue indicates that current discharge is above EFR deficit by 1–100 km^3^ month^−1^). (Online version in colour.)

**Figure 3.**
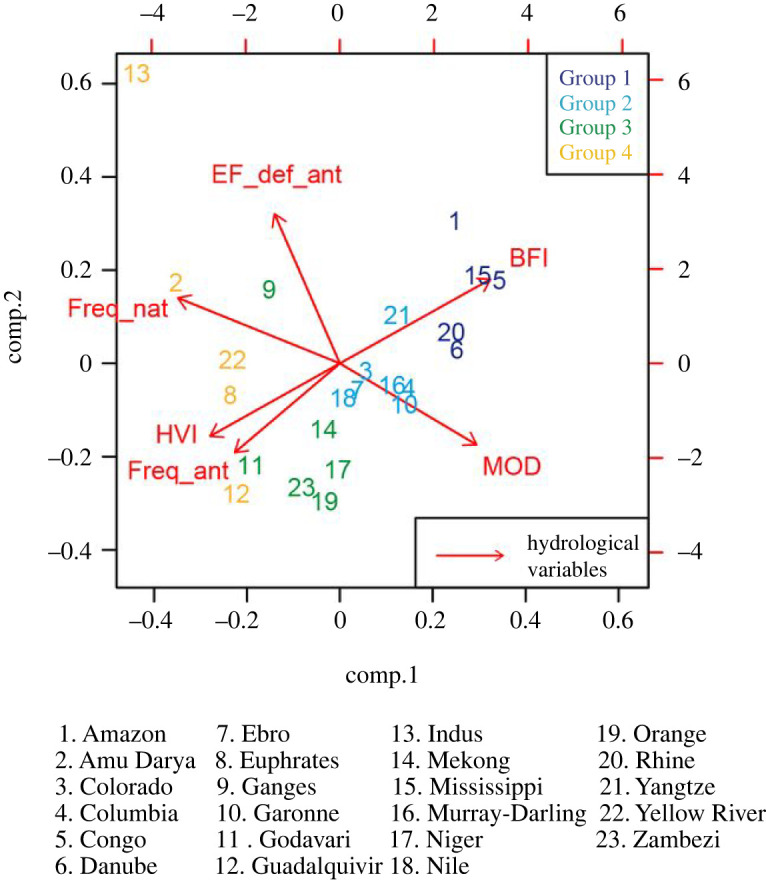
Principal Component Analysis of 23 river basin using six hydrological variables: EF_def2 (Total deficit), Freq_nat (Natural deficit), Freq_ant (Anthropogenic deficit), BFI (Baseflow index), HVI (hydrological variability index) and MOD (the rate of flow alteration). (Online version in colour.)

Our simulations show that the total annual discharge is about 44 000 km^3^ per year, of which EFRs represent between 18 000 and 28 450 km^3^ year^−1^ (40–60% of the global discharge), depending on the EFRs threshold chosen. We show that the total EF deficit represents less than 5% of the total global discharge and ranges between 1068 and 2075 km^3^ yr^−1^ (figures 1 and 2; table 1). That result shows that freshwater planetary boundaries are transgressed. We also show that the anthropogenic deficit from surface waters represents between 260 to 545 km^3^ yr^−1^ (about 25% to 50% of the total anthropogenic deficit). The total annual deficit can be significant at specific temporal and regional scales (figure 3). For example, the highest monthly deficits are observed in South and East Asia (up to 80 km^3^ month^−1^) with a duration of at least five months (between December and May). Among them, the Indus River shows the maximum annual deficit (200 km^3^ per year representing 130% of total discharge). The frequency of the deficit can reach up to 90% of the time (figure 3, supplementary table S2 ) indicating that, in the Indus, monthly flows meet EFRs only 10% of the time. North African and Mediterranean river basins also show large deficits, particularly during the dry season. For example, the Euphrates has a deficit of up to 25 km^3^ per month and up to 60 km^3^ per year (the deficit equals 50% of the discharge).

**Table 1.** Global volumes of discharge, EFRs, deficit and irrigation (km^3^ yr^−1^) also expressed as a share of surface water and groundwater withdrawals (%). s.d. refers to standard deviation.

	absolute values (km^3^ yr^−1^)	share of irrigation Wd (%)
discharge (±s.d.)	44 387 ± 1619	—
EFR (+2s.d.)	18 089–28 450	—
irrigation withdrawals (1960–2000)	1088–2075	—
total water deficit (1960–2000)	1068–1353	66–84%
surface water deficit (1960–2000)	260–545	16–34%

### (b) Categorization of water basins by EF deficit

A PCA was performed to categorize four groups of river basins (figure 3, electronic supplementary material, table S2). The results of the PCA allowed the definition of four river categories using the BFI and the MOD variables:

1. Group 1: Perennial flow regime with low flow modification: BFI ≥ 0.3 and MOD > 0.95
2. Group 2: Perennial/intermittent flow regime with low flow modification: 0.15 ≤ BFI < 0.3 and MOD > 0.7
3. Group 3: Perennial/intermittent flow regime with moderate flow modification: 0.01 ≤ BFI < 0.15 and MOD > 0.7.
4. Group 4 : Perennial/intermittent flow regime with high flow modification: 0.01 ≤ BFI < 0.15 and MOD ≤ 0.7.

The location of the river basins is presented in electronic supplementary material, table S1 and figure S1. The PCA is defined by two components that explain 79% of the variance of the dataset representing the river basins. Six out of 16 hydrological variables were selected when the maximum variance explanation was reached. The first component explains 52% of the variance which is characterized by the BFI and the MOD variables on the positive axis (+0.4) and by the frequency of the natural deficit (Freq_nat), HVI and the frequency of the anthropogenic deficit (Freq_ant) on the negative side of the axis (up to −0.4). The second component is explained by the anthropogenic EF deficit (EF_def_ant) as a positive value (up to 0.4) and the Freq_ant and MOD values as negative values (up to −0.2). We note that the river basins groups follow component 1 with groups starting from the least altered flow (group 1) up to the most altered flow (group 4).

### (c) Transgression of water PB for group 1: perennial rivers with low EF deficit

**Figure 4.**
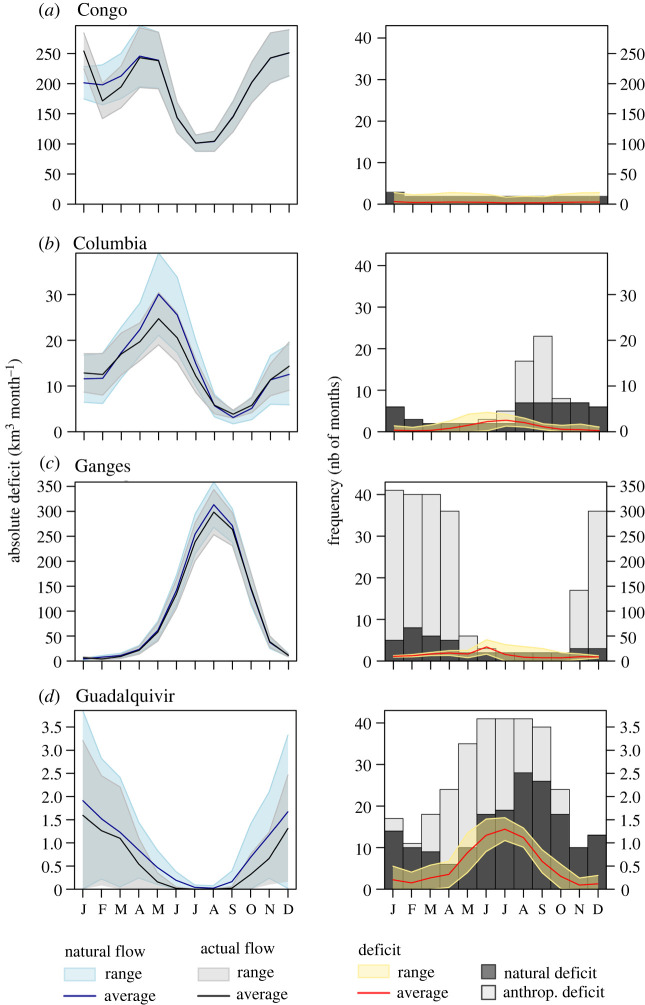
Temporal representation of natural and actual flow (left plots) with the intra-annual monthly deficit (right plots) in km^3^ month^−1^. The frequency of deficit is presented by the number of months in the barplots (right plot) for four river basins representing each group of river basins. Y-axis representing absolute flow and deficit have the same scale. Bar plots represent the frequency of water deficit due to climate (black colour) and due to water abstraction (grey colour). Total environmental deficit (EF) (yellow range-right chart) was shown with positive values for comparability with the flow (blue/grey range – left plot). (Online version in colour.)

Group 1 is located in the axis of the variable BFI (Amazon, Congo, Mississippi, Rhine, Danube). This group is characterized by rivers with low flow variability: BFI greater than 0.38 and HVI less than 0.76 (electronic supplementary material, table S2; figure 4*a* and electronic supplementary material, figure S2). This group is dominated by perennial rivers of which the deficit is mainly natural (25–45 months out of 492 months) versus anthropogenic deficit (0 to 87 months out of 492 months). Group 1 has also the smallest deficit to discharge (DTD) ratio (2–12%). The ratio of the anthropogenic deficit to discharge represents 0 to 7% of the discharge and the ratio of the deficit to withdrawals (DTW) is up to 27% of withdrawals (e.g. Rhine). For example, the Congo River has the most stable flow regime (BFI = 0.54, HVI = 0.49) and shows barely any flow modification except between January and March. Finally, the Congo River is characterized by a low deficit frequency (2 years out of 41) without any anthropogenic deficit.

### (d) Transgression of PB for group 2: perennial rivers with medium EF deficit

Group 2 is located along the axis of the MOD variable (Yangtze, Columbia, Garonne, Colorado, Nile). This group is characterized by less stable flows (BFI = 0.1–0.27 and HVI = 0.74–0.97) (electronic supplementary material, table A2; figure 4*b* and electronic supplementary material, figure S2). The frequency of the deficit is moderate with 78 to 184 months of deficit out of 492 months of which 37 to 60% is due to the anthropogenic deficit. The DTD represents between 7 and 24% (e.g. Ebro) and anthropogenic deficit, 3 and 13% of total discharge. Potentially affected withdrawals (deficit to withdrawals) represent 34% to 43% of withdrawals. Potentially affected withdrawals from surface water can reach up to 31%. For example, the total simulated flow of the Columbia River equals 171 km^3^ yr^−1^, of which 8% is withdrawn for irrigation and other users. The total deficit equals 12 km^3^ (7% of total discharge), of which the natural deficit represents 63% of the total deficit and occurs between August and February. The anthropogenic deficit is 3% of the total flow and has a frequency of 31 months out of 492 (between June and October). Potentially affected water withdrawals represent up to 32% (of which 20% affect surface water withdrawals).

### (e) Transgression of PB for group 3: intermittent rivers with medium EF deficit

Group 3 is characterized by the variables Freq_ant and MOD and includes the following rivers: Godavari, Niger, Zambezi, Orange, Mekong. This group represents a cluster of river basins with high seasonality and with a moderate flow modification (BFI = 0.01–0.05, HVI = 1.14–1.92) (figure 4*c* and electronic supplementary material, figure S32 and table S2). The frequency of deficit is 95 to 285 months out of 492, and 18% to 58% of the deficits are anthropogenic. The DTD ranges from 2 to 17% (e.g. Godavari). Potentially affected withdrawals represent 28 to 72% of total withdrawals (4–66% for withdrawals from surface water only). For example, the Ganges has an intermittent flow regime with a BFI of 0.05 BFI and HVI of 1.69. Withdrawals represent 186 km^3^ (15% of total flow). The total deficit equals 142 km^3^ yr^−1^ (11% of total flow) and is spread over 227 months (less than 50% of the time) of which 185 months are due to the anthropogenic deficit (81% of total deficit). The duration of the deficit is about four months (between November and April), and potentially affected withdrawals represent 62% (of which 26% affect surface water withdrawals).

### (f) Transgression of PB for group 3: intermittent rivers with high EF deficit

Group 4 is highly correlated with EF_def_ant (especially the Indus), with Freq_nat (Amu Darya) and with HVI and Freq_ant (Yellow river Euphrates and Guadalquivir). In group 4, a deficit occurs at least half of the time. Significant deficits are also observed in the western part of the United States such as in Colorado and the Columbia rivers. Finally, perennial rivers, such as the Congo and the Amazon, show a very low EF deficit (less than 2% of the total discharge) resulting mainly from the natural deficit and occurring less than 5% of the time. This group represents a set of river basins with intermittent to ephemeral flow regimes and with a high flow modification (BFI = 0.01–0.11, HVI = 1.15–1.62, MOD = 0.22–0.7) (electronic supplementary material, table A2; figure 4*d* and electronic supplementary material, figure S2). The frequency of deficit is 286 to 461 months out of 492 months, and 42% to 85% of the deficit is anthropogenic. The DTD ranges from 49 to 130% (for the Indus). Potentially affected withdrawals represent 41 to 66% of total withdrawals (of which 8–36% affect only surface waters). For example, the Guadalquivir has an intermittent flow regime with 0.01 BFI and 1.28 HVI. Withdrawals represent 7 km^3^ (66% of total flow). Total deficit equals 6 km^3^ yr^−1^ (64% of total flow) and is spread over 314 months (64% of the time) of which 133 months are due to the anthropogenic deficit (42% of total deficit). The duration of the deficit is about six months (between May and October) and potentially affected withdrawals represent 41% (of which 8% affect surface water withdrawals). The remaining river basin deficits are shown in electronic supplementary material, figure S3.

### (g) Analyses of frequency and timing of PB transgression by river basin group

We show that the higher the BFI, the lower the frequency of natural deficit (*R*^2 ^= 0.35). This means that perennial rivers encounter less deficit due to climate variability than intermittent rivers. Similarly, the BFI is negatively correlated with the frequency of anthropogenic deficit (*R*^2 ^= 0.32), meaning that intermittent rivers encounter more deficits due to anthropogenic sources than perennial rivers.

**Figure 5.**
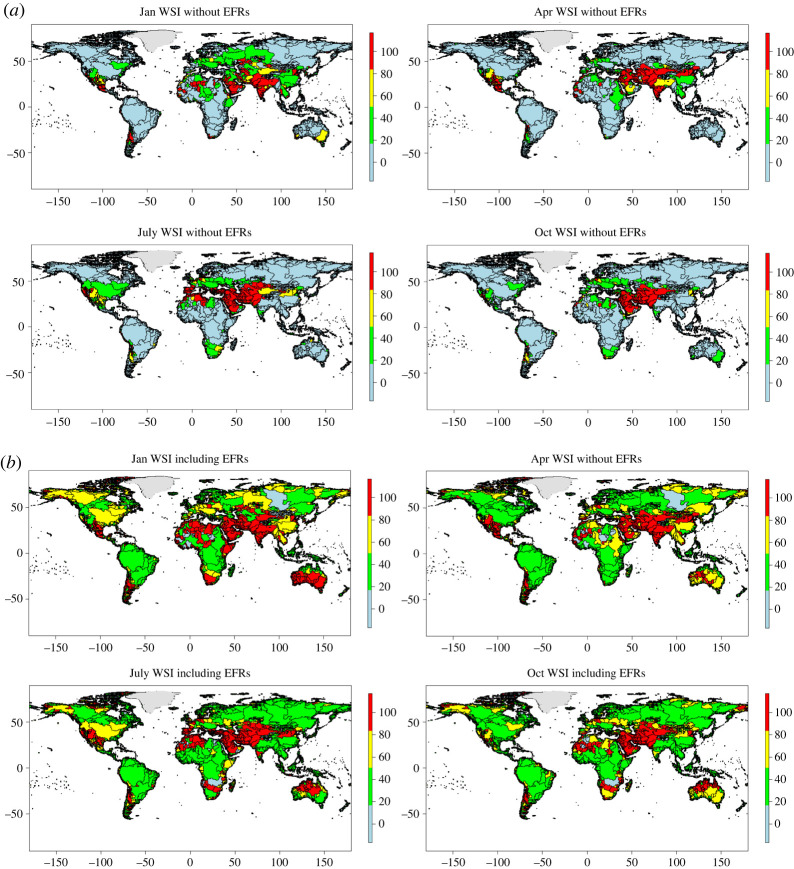
Spatial representation of monthly water stress index (WSI) for January, April, July and October on the global scale without EFRs (*a*) and considering EFRs (*b*) averaged over 41 years aggregated on a river basin level. Units are in percentage of water withdrawn per water available. Levels of WSI are defined as such: low in blue (between 0 and 20%), medium in green (20% to 45%), high in yellow (45% to 80%) and scarce in red (above 80%). (Online version in colour.)

We present one river basin per group in figure 5, showing the magnitude of natural and actual monthly flows (left chart) and the magnitude and frequency of natural and anthropogenic deficits (right chart). The remaining river basins and their respective deficits are shown in electronic supplementary material, figure S2.

Group 1 is represented by the Congo River (figure 4*a*) with a low modification of the natural flow (in blue) compared to the actual flow (in grey). The absolute monthly deficit is below 15 km^3^ and the frequency of the monthly deficit is low (below five months out of 41). In this group, the total deficit is due solely to climate variability.

Figure 4*b* shows the flow dynamics of the Columbia River representing group 2. The range of anthropogenic flow was reduced compared to the natural flow, and the actual flow is below the natural flow from April to August. The absolute monthly deficit is about 5 km^3^ with strong summer deficits (in June and July). The frequency of the monthly deficit is less than 10 months out of 41 for the natural deficit and the anthropogenic deficit frequency occurs mainly between August and September (up to 24 months out of 41). The frequency of the natural deficit is spread over six months and the anthropogenic deficit is spread over three months. The total deficit to withdrawals represents 32% of which 20% impact only surface waters.

Group 3 is represented by the Ganges (figure 4*c*) showing a high flow modification compared to the actual flow. The absolute monthly deficit reaches up to 5 km^3^ in June (31–50% of annual discharge). The total frequency deficit is nearly 100% between December and April where the anthropogenic deficit frequency represents more than 80% of the total deficit frequency. The total deficit to withdrawals represents 62%, of which 35% impact only surface waters.

Finally, group 4 is represented by the Guadalquivir River, which has an actual flow lower than natural flow throughout the year (figure 4*d*). The total monthly deficit can reach up to 20 km^3^ in June-July and exceeds the natural and actual flows during the summer months. The frequency of total deficit occurs every month with at least 20 out of 41 months of deficit. The frequency of natural deficit is between 10 and 30 months out of 41 with peaks in late summer. The frequency of the anthropogenic deficit is about 10 to 25 months out of 41 occurring mainly between April and September. The Guadalquivir River has a total deficit of about 64% of the total discharge. The total deficit to withdrawals represents 66%, of which 15% affect only surface waters.

### (h) Analysis of global water stress considering spatially integrated EFRs

Figure 5 and electronic supplementary material, figure S4 present the water stress indicator (WSI) at the global scale, with and without the inclusion of EFRs. Without EFRs, regions with a low WSI (value less than 40%) encompass the Baltic, Latin America, Europe, Sub-Saharan Africa, the Pacific Islands and Oceania. In January, countries of Asia and around the Equator belt most often show a high WSI. On the other hand, in July, we show that countries from the Middle East and North-African (MENA) mainly are affected by water stress. These two periods correspond to the dry season and are usually marked by high irrigation requirements. When EFRs are taken into account, WSI increases by more than 20% (electronic supplementary material, figure S5), and with the same spatial distribution of water stress than without EFRs (figure 5 and electronic supplementary material, figure S4). While Latin America, North America, Oceania and SSA present low to moderate water stress, other regions in Asia, MENA and in the Commonwealth of Independent States (CIS) experience seasonal water scarcity (WSI > 80%).

## 4. Discussion

### (a) General result overview

We provide a global analysis of classifying patterns of freshwater planetary boundary transgressions at the global scale and among 23 river basins. The analysis tentatively indicates ways to manage or avoid these patterns. Compared to the indicator as defined by [50], it now provides integrated spatially disaggregated EFRs to the global water stress indicator. For the EF deficit, we identified the frequency of the deficit over 41 years (inter-annual variability), the timing, the duration of the deficit (intra-annual variability), and in which group of river basin the deficit was more frequent. We used the VMF method to calculate EFRs monthly and at a spatial resolution of 0.5°; then aggregated to the river basin scale and to the country scale. This is the first study showing differences between natural and anthropogenic monthly EF deficits, including intra- and inter-annual variabilities. We showed that the total EF deficit represents a low share of the global discharge (less than 5%). However, at a regional scale, we demonstrated that the monthly deficit can reach a significant share of the flow or even exceed the available flow (usually during low-flow periods) in locations with high irrigation abstractions. Correlations were found between river flow regime and the level of flow alteration. For example, tropical perennial flow regimes such as the Amazon and the Congo tend to have low EF deficits with a high proportion coming from natural deficit, while intermittent flow regimes in dry areas (and usually requiring high irrigation demand) show a high absolute deficit with a high proportion coming from the anthropogenic deficit and occurring more than 50% of the time.

### (b) Relevance of the EF deficits to PB transgressions

In the study, we show that EF deficit was below 5% of total discharge, about 2200 km^3^ yr^−1^, which is below the PB value of Steffen but relatively close. In terms of regional PB transgression we show similar patterns as in [11,18], except that in our study we also show the frequency of months with climate and anthropogenic deficit. In our study, we also provide the EF deficit globally and per river basin to represent water PB boundaries. By differentiating the climatic variability to the deficit due to water use, we allow water managers and international laws to target their solutions toward sustainable water use and/or climate adaptation solutions. Also, depending on the type of river (perennial or intermittent) and its level of abstraction (low, high), it is easier to decide whether intervention should be focused on preserving the type of flow (low, medium, high) and/or restoring or preserving initial water discharge.

### (c) Relevance of studying EF deficit due to climate variability

Our results showed that anthropogenic and natural deficits are usually combined and seldom independent because water demand for agriculture (e.g. evapotranspiration and irrigation) is usually higher during droughts. Only in the case of perennial tropical rivers with high flows such as the Amazon and the Congo, natural deficit accounted for most of the deficit. The natural deficit is usually related to hydrological droughts that are responses to meteorological droughts. According to Keyantash and Dracup [51], the response of a hydrological drought (and natural deficit) depends on the location of the climatic drought and the time it takes for the flow to reach downstream areas. For example, in the Amazon River, we found that drought years and natural deficit are occurring in the same years (1973, 1982 and 1991), linked to El Niño events [52]. Furthermore, meteorological droughts leading to higher evapotranspiration and irrigation requirements can exacerbate the anthropogenic deficit [53].

### (d) Limits of our study

In this study, we have calculated water flows at 0.5 deg. and have aggregated these fluxes to the river basin level (more than 11 000 river basins) and to the country level (91 countries). We show our results with the spatial resolution of the river basin since it is a better spatial resolution than the country level. It is important to note that the maps presented here are only relevant to understand the regional hotspots of water stress and EF deficit in space and time and that specific evaluation at the hydrograph level needs to be carried out to understand the hydrological patterns and the cause of potential water stress and EF deficit. This is why we have zoomed to 23 major river basins. In order to look at other potential EF deficit and/or regional PB transgression, other studies have done similar analyses such as in [18].

In this study, regions with high levels of water stress were characterized by the percentage of water abstraction-to-availability, which is spatially and temporarily highly variable. Hot spots of the MENA and South-Asian regions were found to have consistent high water stress in terms of magnitude and frequency, as in Kummu *et al*. [54]. They show that global water consumption and water stress have increased fourfold since the beginning of the twentieth century. Therefore, solutions to restore and/or protect rivers should be targeted to a specific group of rivers, and the type of pressure and EF deficit should be associated. For example, Mediterranean areas are subject to both natural and anthropogenic deficits due to long dry seasons combined with high irrigation abstractions. Tackling these two different types of deficits might require different measures, e.g. for natural deficits, it may be necessary to increase water storage [55], and for anthropogenic deficits, to decrease the production of water-intensive crops [56]. In this study, we show that surface water withdrawals for irrigation are responsible for 16–34% of the anthropogenic deficit and this is also shown in Jägermeyr *et al*. [11] where it is shown that if 14% of the irrigated production was lost, food security would be at risk. Similarly, other studies show that reducing water use for irrigation to preserve ecosystems can lead to water-related conflicts among users [57,–59]. A specific example is shown in Blanco-Gutiérrez *et al*., [60] where compliance with the Water Framework Directive in the Guadiana River would reduce the use of water for irrigation and would affect rice growers. Another study shows the importance of estimating the deficit according to its flow regime and flow alteration, as in the example of the Segura River. This example is characterized by an intermittent river with high water abstractions where four levels of adaptation to drought management have been defined: normal, pre-alert, alert and emergency. The resulting irrigation withdrawals from surface water were respectively reduced by 100%, 90%, 75% to 50%. This type of drought management has proven to be effective when groundwater rights have been defined [53]. Finally, payments for river ecosystem services, such as natural water purification, erosion control, fish and wildlife habitat and recreation, have provided good incentives to limit water stress in some communities [61].

### (e) Potential policies and solutions to improve water availability

To protect rivers, it is necessary to improve environmental monitoring of rivers and to anticipate responses to flow changes by selecting appropriate methods [62]. For example, our results show that irrigation abstractions might need to be reduced to meet EFRs, especially in modified intermittent rivers, and that specific flow releases from storage could be applied such as stabilization ponds (lagoon systems) from waste water treatment plant to meet EFRs and preserve ecosystems [55]. Additionally, decision-support models and hydro-economic models could help meet both irrigation water requirements and EFRs [60,63,64]. Solutions to save water locally and other water demand and supply measures should be included in future studies and assessments, such as increasing green water storage, purifying greywater, reducing virtual water export [65], increasing water use efficiency and desalinating brackish water [66]. Attention should also be paid to ensure return flows to recharge groundwater and downstream rivers [64,67]. Another option to decrease local and regional deficits in water-scarce regions would be to decrease irrigation via an increase in food imports from water-abundant regions and via the use of more dam regulation [65]. However, these solutions should not come at the expense of local food security and/or increased environmental degradations.

### (f) Further research and potential limits of our study

In this study, we examined the cause of the EF deficit and defined the timing, volume, duration and frequency of the deficit for 23 river basins. The concept of EF deficit is a step forward in the field of eco-hydrology, providing key hydrological tools to water managers and freshwater ecologists regarding when, where, and why freshwater ecosystems are at risk. Xenopoulos *et al.* [68] have shown that flow alteration could be linked with the loss of freshwater species richness. Similarly, Pracheil *et al*. [69] were able to define flow thresholds that maintain most specialized species of the Mississippi River. However, further research is needed on ‘how the trait composition of stream communities varies along geographical and environmental gradients' [70]. The use of seasonal and supra-seasonal EF deficit definitions could be useful for monitoring streams and adopting the most appropriate alternative. Future research could also address the effects of climate change, land-use change and future socio-economic scenarios to evaluate the intensity, duration, frequency and spatial distribution of deficits under global change. As future climate is likely to be more variable due to intensification of the hydrological cycle, this could exacerbate pressure on water supply for humans and ecosystems [62,71]. For example, a study from Rajagopalan *et al*. [72] showed that a 20% reduction in the Colorado River flow would imply a 10-fold reduction in reservoir storage. Including parameters other than flow alteration in eco-hydrological assessments could also be beneficial, such as including changes in water temperature or water quality [73]. It has been shown that future water temperature is likely to increase in the US, Europe and China and will likely be exacerbated by decreasing low summer flows [74].

## 5. Conclusion

This study presents a new concept for calculating transgressions of the freshwater planetary boundary. We evaluated water deficit in terms of duration, timing and frequency for the world's major river basins. Environmental flow deficit was found to be a regional rather than a global concern (globally, EF deficits account for less than 5% of total discharge). The study highlights the most affected regions, such as South Asia, the Mediterranean and Middle East area and the West Coast of the US, where immediate action is needed to restore river flows and avoid further degradation of riverine ecosystems. In this study, we also identified the causes of EF deficit (natural and anthropogenic). This allows for greater specificity in the choice and level of intervention of water managers. We found correlations between the cause of deficit (natural or anthropogenic), the level of flow modification (in magnitude) and the type of flow regime (perennial or intermittent). Intermittent rivers with moderate to high flow alterations are likely to encounter anthropogenic deficits. We show that targeted actions may be required in heavily altered rivers where EF deficit exceeds the available flow. Special attention is required for future water allocation in pristine rivers and in river basins with current low EF deficit to avoid stream degradation. Finally, the inclusion of EFRs shows a higher water stress index globally, ranging from 30% in temperate zones to extremely high WSI values in MENA and Southeast Asia [75,–76].

## Data Availability

Data and materials are available upon request from the authors. The datasets supporting this article have been uploaded as part of the electronic supplementary material. The data are provided in electronic supplementary material [75].
